# Prediction of interactomic hub genes in PBMC cells in type 2 diabetes mellitus, dyslipidemia, and periodontitis

**DOI:** 10.1186/s12903-024-04041-y

**Published:** 2024-03-26

**Authors:** Pradeep kumar yadalam, Deepavalli Arumuganainar, Vincenzo Ronsivalle, Marco Di Blasio, Almir Badnjevic, Maria Maddalena Marrapodi, Gabriele Cervino, Giuseppe Minervini

**Affiliations:** 1 Department of periodontics, Saveetha Institute Of Medical And Technical Science (SIMATS), Saveetha Dental College and Hospital, Saveetha University, Chennai, India; 2 Department of Periodontics, Ragas Dental College and Hospital, Chennai, India; 3 Department of Biomedical and Surgical and Biomedical Sciences, Catania University, Catania, 95123 Italy; 4 Department of Medicine and Surgery, University Center of Dentistry, University of Parma, Parma, 43126 Italy; 5 Verlab Research Institute for Biomedical Engineering, Medical Devices, and Artificial Intelligence, Bosnia and Herzegovina, Sarajevo, 71000 Bosnia-Herzegovina; 6 Department of Woman, Child and General and Specialist Surgery, University of Campania “Luigi Vanvitelli”, Naples, 80121 Italy; 7 School of Dentistry, Department of Biomedical and Dental Sciences and Morphofunctional Imaging, University of Messina, via Consolare Valeria, 1, Messina, 98125 Italy; 8 Saveetha Dental College and Hospitals, Saveetha Institute of Medical and Technical Sciences (SIMATS), Saveetha University, Chennai, Tamil Nadu India; 9 Multidisciplinary Department of Medical-Surgical and Dental Specialties, University of Campania “Luigi Vanvitelli”, Caserta, 80121 Italy

**Keywords:** Bioinformatics, Chronic periodontitis, Dyslipidemia, Hub gene, Immunity, Inflammation, Peripheral blood mononuclear cells, Type 2 diabetes mellitus

## Abstract

**Background and objective:**

In recent years, the complex interplay between systemic health and oral well-being has emerged as a focal point for researchers and healthcare practitioners. Among the several important connections, the convergence of Type 2 Diabetes Mellitus (T2DM), dyslipidemia, chronic periodontitis, and peripheral blood mononuclear cells (PBMCs) is a remarkable example. These components collectively contribute to a network of interactions that extends beyond their domains, underscoring the intricate nature of human health. In the current study, bioinformatics analysis was utilized to predict the interactomic hub genes involved in type 2 diabetes mellitus (T2DM), dyslipidemia, and periodontitis and their relationships to peripheral blood mononuclear cells (PBMC) by machine learning algorithms.

**Materials and Methods:**

Gene Expression Omnibus datasets were utilized to identify the genes linked to type 2 diabetes mellitus(T2DM), dyslipidemia, and Periodontitis (GSE156993).Gene Ontology (G.O.) Enrichr, Genemania, and Kyoto Encyclopedia of Genes and Genomes (KEGG) pathways were used for analysis for identification and functionalities of hub genes. The expression of hub D.E.G.s was confirmed, and an orange machine learning tool was used to predict the hub genes.

**Result:**

The decision tree, AdaBoost, and Random Forest had an A.U.C. of 0.982, 1.000, and 0.991 in the R.O.C. curve. The AdaBoost model showed an accuracy of (1.000). The findings imply that the AdaBoost model showed a good predictive value and may support the clinical evaluation and assist in accurately detecting periodontitis associated with T2DM and dyslipidemia. Moreover, the genes with p-value < 0.05 and A.U.C.>0.90, which showed excellent predictive value, were thus considered hub genes.

**Conclusion:**

The hub genes and the D.E.G.s identified in the present study contribute immensely to the fundamentals of the molecular mechanisms occurring in the PBMC associated with the progression of periodontitis in the presence of T2DM and dyslipidemia. They may be considered potential biomarkers and offer novel therapeutic strategies for chronic inflammatory diseases.

## Introduction

In recent years, the intricate connections between systemic health and oral well-being have garnered significant attention among researchers and healthcare practitioners. One such multifaceted interrelation exists at the crossroads of Type 2 Diabetes Mellitus (T2DM), dyslipidemia, and chronic periodontitis [1]. Each condition substantially impacts health, with consequences extending beyond their respective domains.

Type 2 Diabetes Mellitus(T2DM), a metabolic disorder characterized by hyperglycemia resulting from insulin resistance and inadequate insulin secretion, has become a global epidemic [2]. Beyond its immediate effects on glucose metabolism, T2DM has been implicated as a systemic inflammatory state, influencing various organs and systems throughout the body [2]. One of the less explored yet critical aspects of this relationship is the potential bidirectional link between T2DM and chronic periodontitis, a chronic inflammatory condition affecting the supporting structures of teeth. This connection may create a cyclical worsening of both conditions, complicating disease management [3–11].

Dyslipidemia, characterized by abnormal lipid profiles in the bloodstream, is another prevalent condition with systemic implications. The disrupted lipid balance often seen in dyslipidemia can contribute to atherosclerosis and cardiovascular diseases [12]. The intricate interplay between dyslipidemia and T2DM and chronic periodontitis can further amplify the systemic inflammatory burden, potentially exacerbating disease progression.

Periodontitis, a prevalent oral inflammatory disease, involves a dysbiotic microbial community interacting with the host immune response, destroying periodontal tissues. Beyond its local consequences, chronic periodontitis has been linked to systemic inflammation and various systemic diseases, including diabetes and cardiovascular diseases [13]. This bidirectional relationship underscores the importance of considering oral health as an integral component of overall health [14–34].

Type 2 Diabetes Mellitus (T2DM), which affects roughly 90% of D.M. patients, is linked to obesity and insulin resistance. D.M. sometimes arises with other systemic abnormalities, such as dyslipidemia, a metabolic inefficiency caused by high blood lipoprotein levels. Dyslipidemia (DL) may contribute to DM-induced immune cell changes. People with diabetes have higher LDL/triglycerides even when blood glucose is effectively managed. Cytokine levels accelerate lipid mobilization from the liver and adipose tissue, which elevates LDL binding to the endothelium and smooth muscles and LDL-receptor gene transcription.T2DM, dyslipidemia, and periodontitis [35, 36] are often found together due to the nonlinear aspect of periodontal disease and diabetes mellitus and the link between periodontitis and reduced lipid metabolism. Identifying hub genes in different diseases is crucial for understanding the underlying molecular mechanisms and pathways. This helps in disease understanding, biomarker discovery, therapeutic target identification, personalized medicine, and network analysis. Hub genes are involved in crucial biological functions and pathways, providing insights into disease biology and potential therapeutic targets. They can also be disease diagnosis, prognosis, and treatment response biomarkers. Hub genes enable targeted treatment approaches by stratifying patients based on molecular profiles.

Machine learning can be used to predict interactomic hub genes, which play a central role in protein interaction networks. Machine learning algorithms can identify and prioritize hub genes based on their potential importance in biological pathways by leveraging network analysis, feature selection, and predictive modeling.

Given the intricate web of interactions between Type 2 Diabetes Mellitus, dyslipidemia, and chronic periodontitis, a comprehensive understanding of their interplay is essential for clinicians and researchers aiming to provide holistic patient care. This study aims at shared pathways to predict hub genes using machine learning in periodontitis and diabetes with dyslipidemia.

## Methods

### Gene expression database

The NCBI GEO dataset [37] GSE156993 was located and downloaded using the keywords periodontitis, peripheral blood mononuclear cells, dyslipidemia, and type 2 diabetes mellitus. The expression patterns of the identified genes were then examined in periodontitis, peripheral blood mononuclear cells, dyslipidemia, and type 2 diabetes mellitus. These databases contained information about gene expression, including which genes were differentially expressed.

### Analyzing differential expression

Differential expression analysis was conducted using appropriate statistical tools to identify the genes that had altered expression. This investigation examines the gene expression levels in T2DM, dyslipidemia, and periodontitis to spot the genes that show substantially significant effects.

### Network analysis

Cytoscape GENEMANIA [38] and the appropriate bioinformatics tools created a gene co-expression network. Network analysis methods were employed to determine the hub genes based on the interactions between the genes and their connectivity within the network.

### Network topology analysis

Genes with high connectivity and central roles were identified using network topology metrics such as node degree, betweenness centrality, and closeness centrality. Hub genes have been found to have substantial effects on network structure and interconnectivity.

### Analysis of genes with differentially expressed functions

Functional Identification of hub genes was done by enrichr for Gene Ontology (G.O.) [39]. The enriched gene sets’ false discovery rate (F.D.R.) was 0.05. A Gene was considered significant if it had at least three genes and a p-value of 0.05 based on t-test analysis between disease and healthy samples. Gene Ontology (G.O.) is a standardized functional annotation system that categorizes genes based on their biological functions, molecular activities, and cellular locations.

### Orange machine learning for predictive hub genes

An open-source toolkit for data visualization and machine learning is called Orange. Orange is a handy tool for data analysis and predictive modeling since it integrates easily with all other well-known machine learning frameworks. Outliers were removed from the top 250 DEGs’ statistical data before subjecting them to machine learning algorithms [40].

The data was divided into training and testing segments using a decision tree, AdaBoost, and random forest widgets, and it was cross-validated to achieve the desired results.

The processes make use of multi-dimensional scaling, model scoring, and cross-validation. Orange integrates Python libraries like NumPy, scipy, and sci-kit-learn into workflow blocks for data manipulation, machine learning method parameters modification, browsing results, and inferred model visualization.

### Decision tree

One non-parametric supervised learning method for classification and regression is decision trees. It has a hierarchical tree structure with internal and leaf nodes, roots, and branches.

### Adaptive boosting

Machine learning ensemble approaches use the AdaBoost algorithm, commonly called adaptive boosting. Adaptive boosting [41] refers to assigning weights to each instance, with heavier weights going to incorrectly identified instances.

### Random forest

**Fig. 1:**
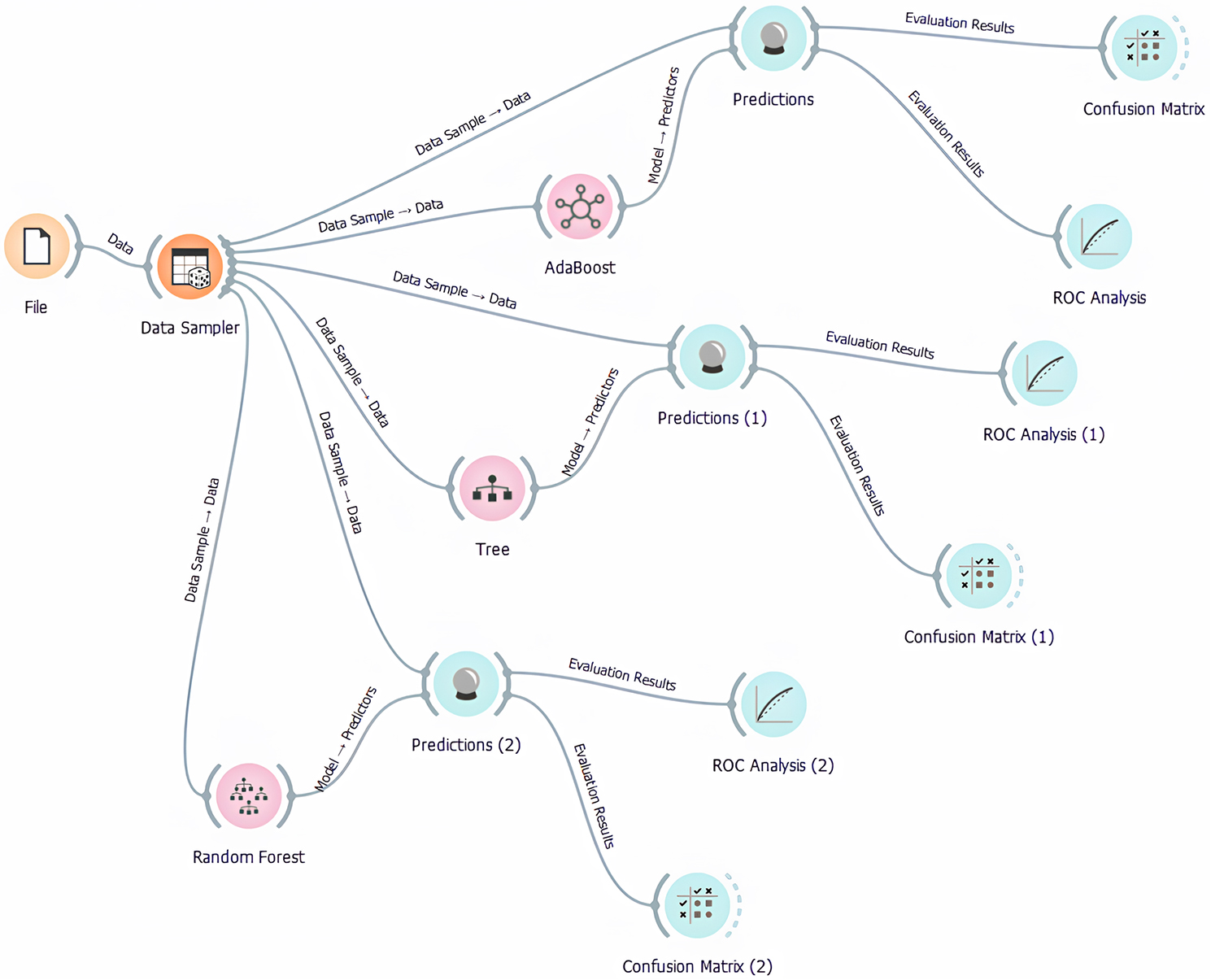
Orange Workflow of the current study

This algorithm for supervised machine learning is well-known. It can be used for ML problems involving both regression and classification. The system learns and determines the output based on the majority decisions of the several decision trees (Fig. 1). The method most frequently used to classify patients based on biomarkers [41].

## Results

**Fig. 2:**
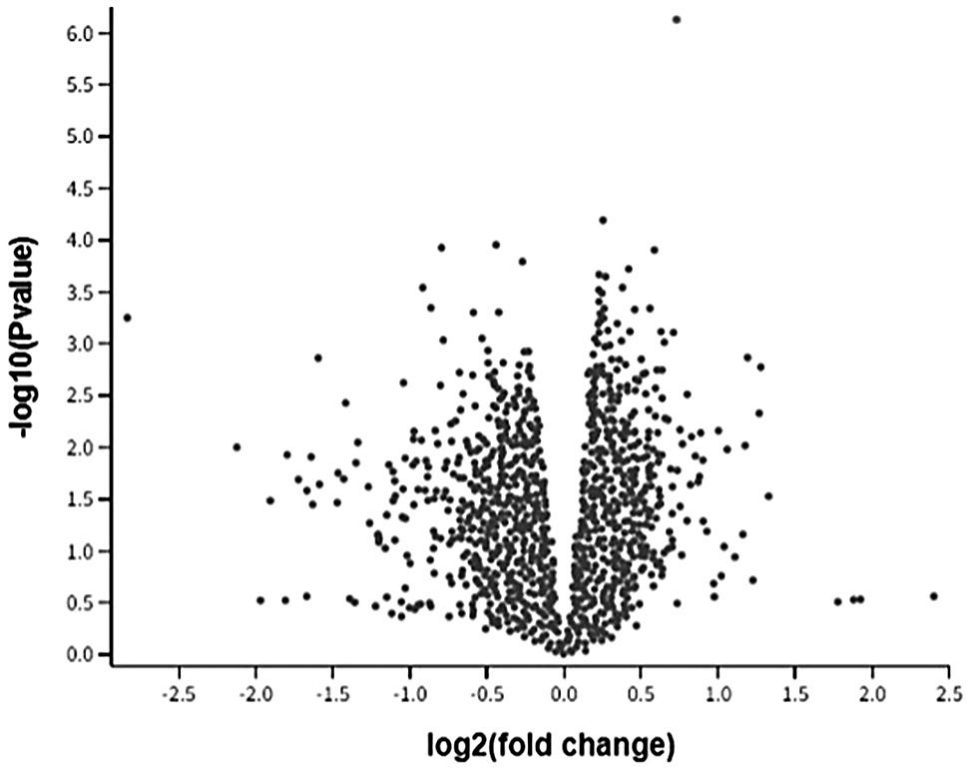
shows a volcano plot of D.E.G.s of PBMCs in periodontitis and diabetes

Gene expression datasets for Periodontitis GSE156993 were chosen from the GEO database. Five hundred differentially expressed genes (D.E.G.s)(Fig. 2) were found using GEO2R from periodontitis. The cut-off criteria used to define D.E.G. were |log2 fold change (F.C.)| >0 and P-value 0.05.

Interactome and Analysis of Differentially Expressed Genes’ Functional Enrichment.

**Fig. 3:**
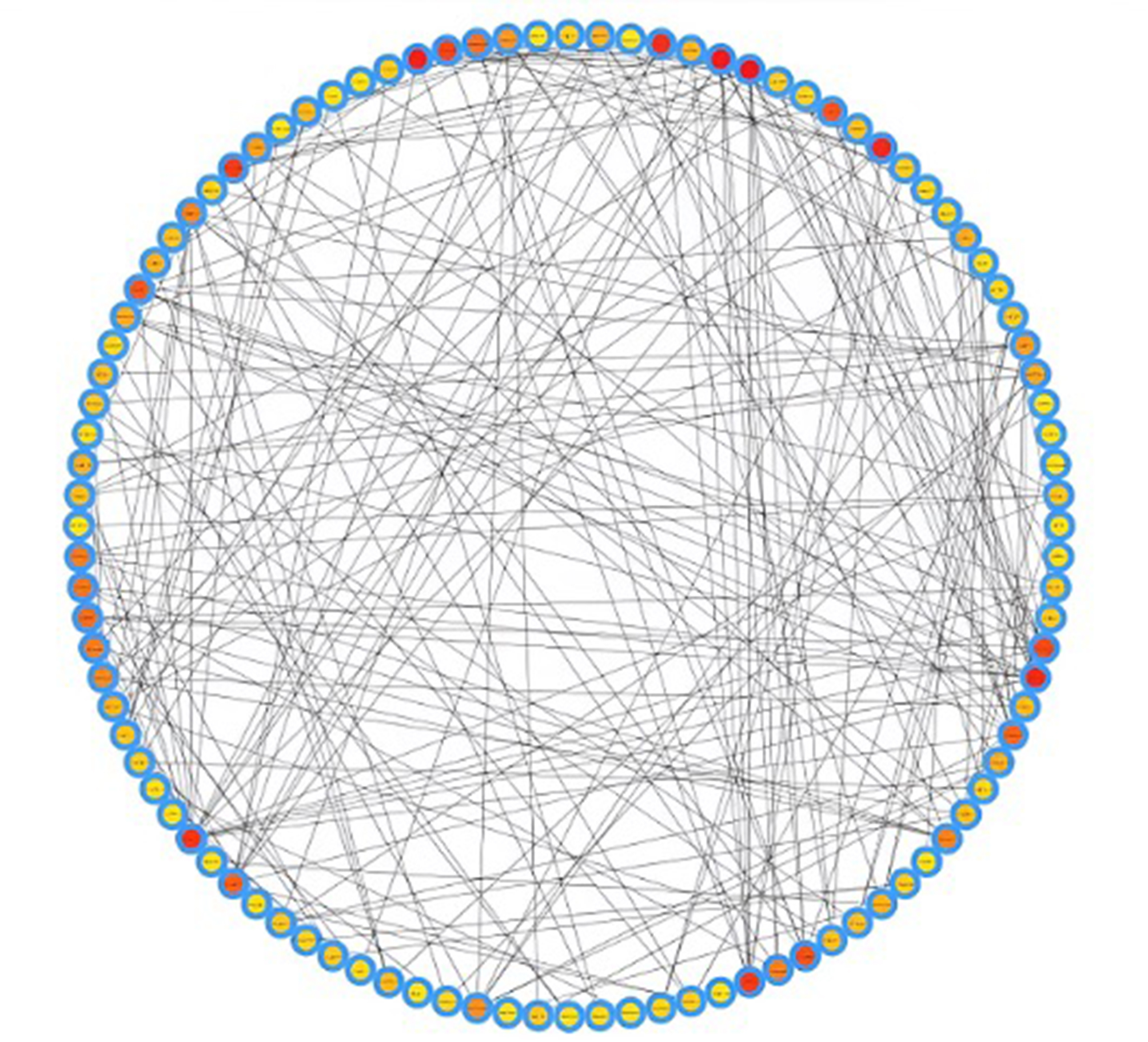
An integrated network of periodontitis, T2DM, and dyslipidemia

**Fig. 4:**
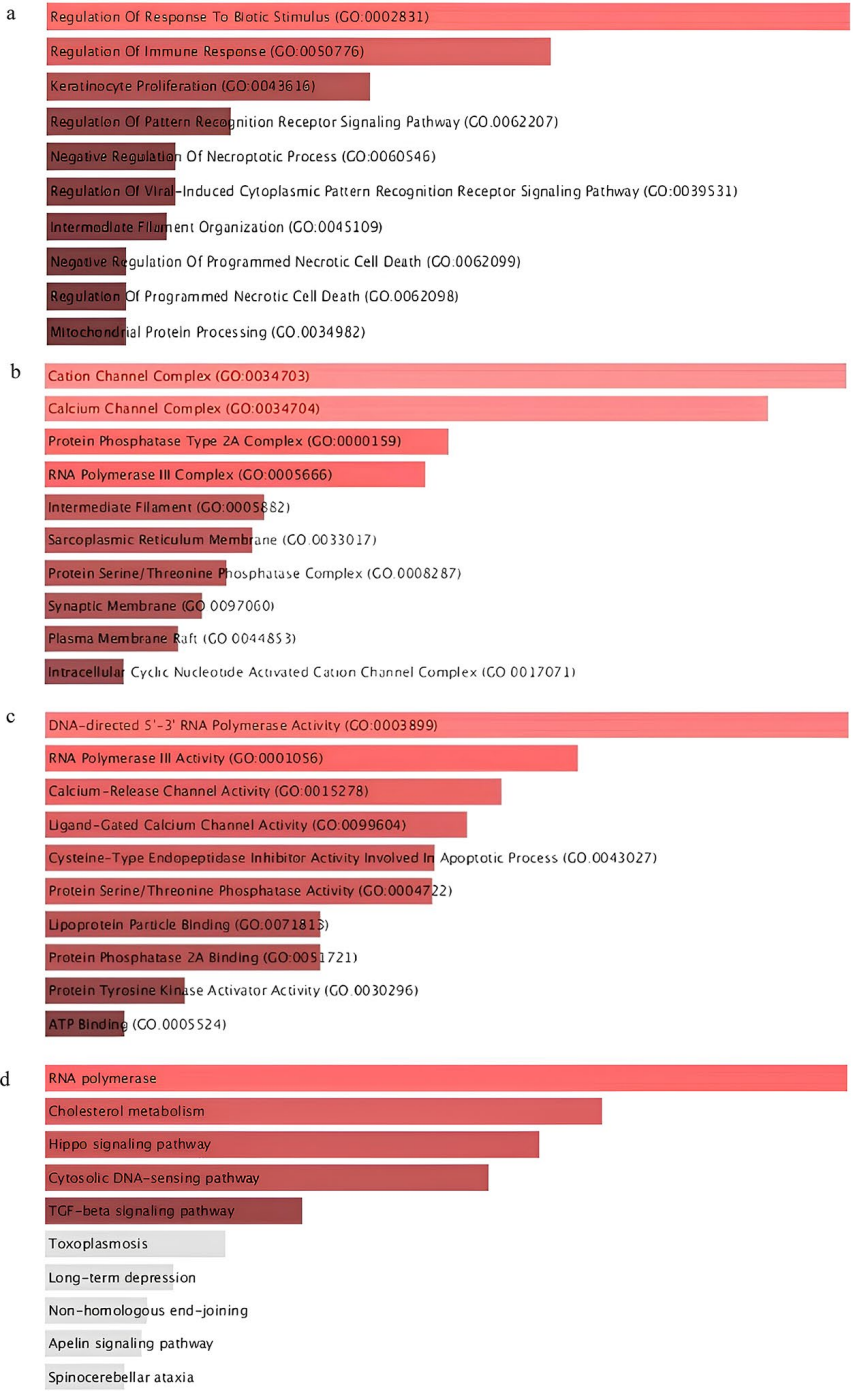
**A** shows the biological function of D.E.G.s in biotic stimulus and keratinocyte proliferation. **B** shows the cellular component cation and calcium channel complex of D.E.G.s. **C** shows the molecular component DNA-directed polymerase activity of D.E.G.s. **D** shows the T.G.F. beta signaling pathway of D.E.G.s

Interactome of periodontitis identified hub genes, and centrality was measured using genemania. (Fig. 3), Gene enrichment analysis was done using Enrichr. According to the G.O. analysis, D.E.G.s are more prevalent in biological processes connected to immunity, including “pattern recognition receptor signaling and “keratinocyte proliferation.” Most of the signaling pathways in D.E.G.s are involved in the immune system, such as the “T.G.F.- beta signaling pathway.” Enrichr was used to do the enrichment analysis. (Fig. 4A to D .)

We recalculated hub gene and non-hub gene similarities in cross-validation steps to assess the effectiveness of predicting interactome hub gene and non-hub gene in periodontitis, diabetes, dyslipidemia, and PBMC associations.

The testing samples’ association likelihood scores are used to organize them. Favorable samples with higher rankings have node connections. Positive samples have a hub gene-disease node pair relationship and an association score above a threshold. To create a receiver operating characteristic (R.O.C.) curve, T.P.R.s and F.P.R.s are calculated. TP/ TP + FN and FP/ FP + TN define TPR and FPR.

**Fig. 5:**
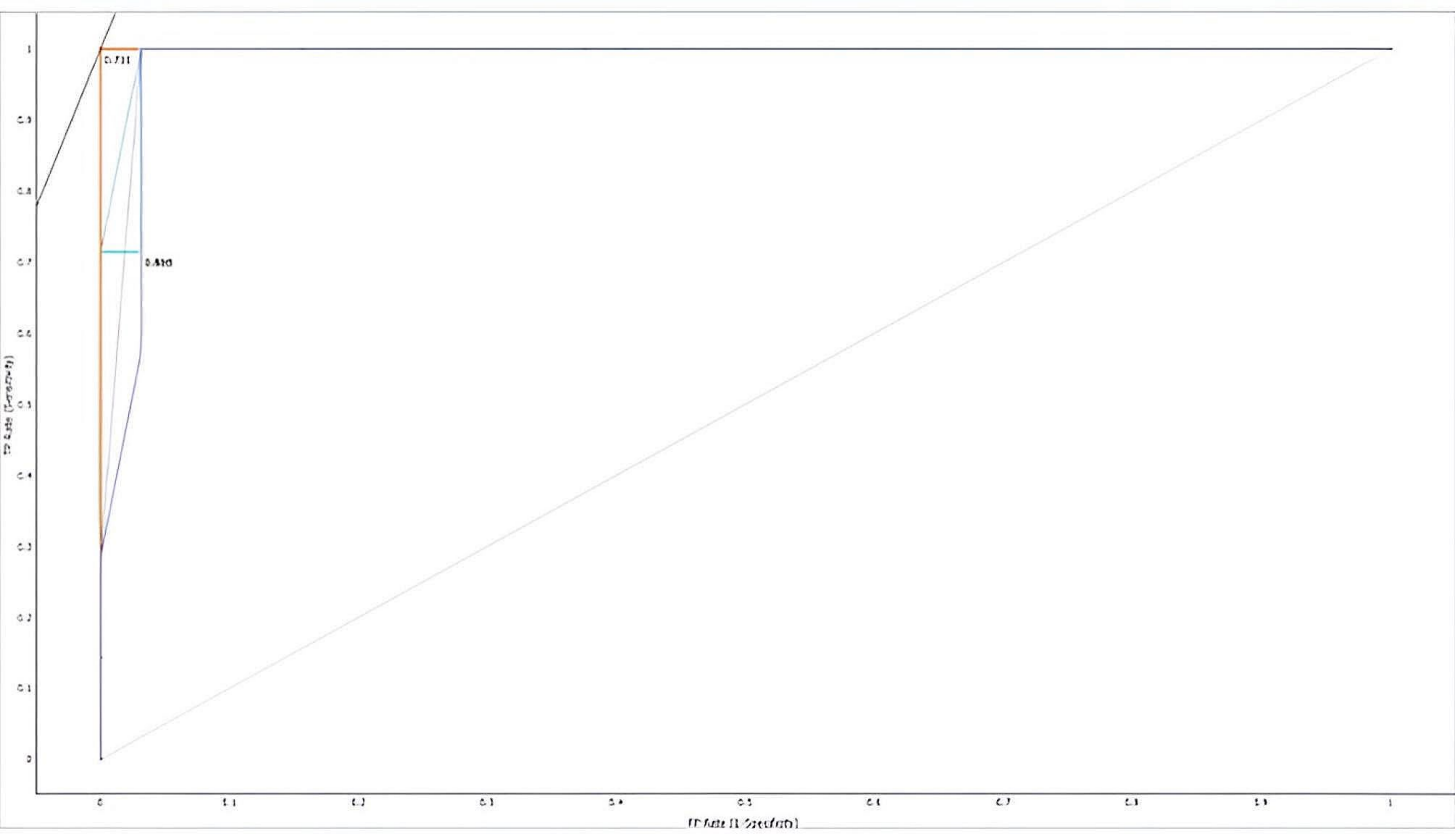
shows the R.O.C. curve for HUB GENE, indicating a good prediction accuracy model

**Fig. 6:**
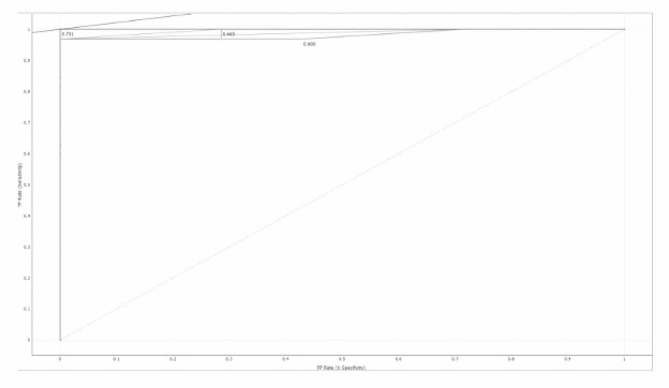
shows the R.O.C. curve Shows a good predictive model for non-hub genes

The sign F.N. denotes the number of hub genes that were mistakenly identified, and the letter T.P. denotes positively identified samples that were successfully recognized. The TN/FP measures the proportion of incorrectly identified positive to properly identified negative samples. A helpful metric for evaluating a method’s overall prediction performance is the area under the R.O.C. curve (A.U.C.). (Figures 5 and 6)

Positive associations between hub gene and non-hub gene disorders are out of proportion with negative correlations. This is when the precision-recall (P.R.) curve and its area (AUPR) are utilized to evaluate a prediction approach. The definitions of recall and precision are as follows:

A good classifier should have one precision (high). Precision only occurs when the numerator and denominator match or when T.P. = T.P. + F.P. F.P. is 0. Thus, this implies. Precision = TP ÷ TP + F.P.

A good classifier must have 1 or 1 recall. T.P. = T.P. + F.N., where F.N. is zero, and recall becomes one if the denominator and numerator are identical. The denominator becomes more important than the numerators as F.N. grows, decreasing recall. Recall = (TP ÷ TP + FN).

Because F.P. and F.N. are zero, a competent classifier has one precision and recall. Therefore, we need a statistic that considers recall and precision. The F1-score, which accounts for precision and recall, is [(Precision x Recall)/(Precision + Recall)] x 2.

AUC-ROC curves can be assessed using a confusion matrix, recall, precision, specificity, and accuracy. The test’s target characteristic is the hub and non-hub gene predictions. The study used stratified sampling with multiple Folds 20 and cross-validation. The matrix indicates how many true positives, true negatives, false positives, and false negatives the model generated from test data.

Results of various models with A.U.C. of 98% for Decision tree, 100% for AdaBoost, and 99% for Random Forest (Table 1).

**Table 1:** Model performance of all algorithms

MODEL	AUC	CA	F1	Precision	Recall	LogLoss	Specificity
Tree	0.982	0.897	0.890	0.879	0.890	0.158	0.498
AdaBoost	1.000	1.000	1.000	1.000	1.000	0.313	1.000
Random Forest	0.991	0.923	0.921	0.920	0.923	0.153	0.760

### Predicted

The decision tree model’s evaluation of the expected outcomes using the confusion matrix produced classification results for the hub gene that were 91.2% for True Positive and 20% for True Negative. (Table 2)

**Table 2:** Confusion Matrix with Decision Tree Model

	HUB	NON-HUB	∑
HUB	91.2%	20.0%	32
NONHUB	8.8%	80.0%	7
∑	34	5	39

### Predicted

When the Adaboost model’s projected results were evaluated, the hub gene’s True Positive prediction was 100%, while the non-hub gene’s True Negative prediction was 0%. (Table 3)

**Table 3:** Confusion matrix with AdaBoost Model

	HUB	NON-HUB	∑
HUB	100%	0	32
NON-HUB	0	100%	7
∑	32	7	39

### Predicted

The classification results for the hub gene True positive were evaluated using the Confusion Matrix on the Random Forest Model. They were 93.9%, whereas the True negative predicted result for the non-hub gene was 32%. (Table 4)

**Table 4:** Confusion matrix with Random Forest Model

	HUB	NON-HUB	∑
HUB	93.9%	16.7%	32
NON-HUB	6.1%	83.3%	7
∑	33	6	39

## Discussion

Interactome hub genes offer insights into biological system complexity, disease mechanisms, and therapeutic targets. They can serve as biomarkers for disease diagnosis, prognosis, and treatment response, enabling personalized medicine and drug development by identifying specific disease conditions. In T2DM, PBMCs may exhibit pro-inflammatory characteristics. There is evidence of increased production of pro-inflammatory cytokines such as interleukin-6 (IL-6) and tumor necrosis factor-alpha (TNF-α) by PBMCs in individuals with T2DM. This inflammatory activation may contribute to chronic low-grade inflammation seen in T2DM [42]. T2DM can lead to impaired immune function, affecting the ability of PBMCs to respond effectively to infections. This can increase susceptibility to various illnesses [43, 44]. In periodontitis, PBMCs may migrate to the periodontal tissues as part of the immune response to the oral infection. This migration can accumulate immune cells in the gingival tissues, contributing to local inflammation.

PBMCs within the periodontal tissues may exhibit an activated pro-inflammatory state, producing cytokines contributing to tissue destruction in chronic periodontitis [14, 45, 46]. Dyslipidemia can affect PBMCs by altering their lipid metabolism. Bloodstream white blood cells are lipid-laden peripheral blood mononuclear cells (PBMCs). They are heavy in fat molecules or lipids. Elevated levels of circulating lipids, particularly low-density lipoprotein cholesterol (LDL-C), can lead to lipid accumulation in PBMCs. This lipid loading can increase oxidative stress and inflammation within these cells [47].

Lipid-laden PBMCs may produce inflammatory mediators, further contributing to systemic inflammation associated with dyslipidemia and increasing the risk of atherosclerosis [48]. Hence, PBMCs in these individuals can produce inflammatory molecules and altered immune responses, which may contribute to chronic inflammation and immune dysfunction, impacting overall health. Both periodontitis and diabetes and dyslipidemia are multifactorial diseases marked by persistent inflammation that results in the deterioration of tissue and bone around the teeth or joints, as appropriate. IGLJ3, DNASE1L3, ABCG1, DPEP2, and KIF19 are highly differentiated genes associated with diabetes and periodontitis. IgLJ3 (Immunoglobulin Lambda Joining 3 [49] is present in higher levels in people with type 1 diabetes than in people without diabetes. This suggests that IgLJ3 may play a role in developing the diseases. DNASE1L3 [50] (Deoxyribonuclease 1-like 3) is an enzyme involved in D.N.A. degradation. It belongs to the deoxyribonuclease I family. Single nucleotide polymorphism can alter protein expression and non-synonymous SNPs, resulting in single amino acid changes affecting protein function and potentially causing disease. Studies have shown that DNASE1L3 levels are elevated in people with diabetes, possibly contributing to diabetic complications.

DNASE1L3- deoxyribonuclease 1 like 3 [51] may contribute to the inflammatory response that destroys periodontal tissue and can cause vascular occlusion in periodontitis. The immune cells secrete the enzyme in response to bacterial infection and break down D.N.A. from bacteria and host cells. This can release inflammatory molecules that injure tissues. Periodontal pathogens have identified changes in cholesterol efflux-related enzymes ( ABCG1- ATP binding cassette subfamily G member one and CYP46A1), contributing to foam cell formation and enhanced Ca2 + signaling and R.O.S. production as critical events in lipid homeostasis disruption. Excess cholesterol ester production via ACAT1 and decreased cellular cholesterol efflux via ABCG1 are two pathways that may contribute to atherosclerosis caused by Pg-LPS [52]. Type 2 diabetics have lower ABCG1 expression and cholesterol efflux. This decreased ABCG1-mediated cholesterol export dramatically increases intracellular cholesterol [53]. ABCA1 dysfunction impairs insulin secretion by disrupting cholesterol transport [54]. Deficit of both ABCA1 and ABCG1 leads to more significant β-cell function abnormalities than either transporter alone, leading to dyslipidemia and diabetes. DPEP2, KIF19 induced diabetes associated with obesity and showing strong interactions with periodontal disease [55].

The present study showed fewer false positives and negatives in the estimated hub genes with ROC. Curve, demonstrating a good predictive model. We need further research with larger sample sizes and improved algorithms to prove that machine learning models are more effective. In this study, adaboost exhibited an AUC of 1 with overfitting, a binary classification model performance metric. Strategies to avoid overfitting include collecting more data, selecting relevant features, regularization techniques, cross-validation, early stopping, ensemble methods, and regular evaluation.

## Conclusion

Predicting interactomic hub genes and deciphering the intricate molecular networks underlying periodontitis and systemic diseases. Novel algorithms can uncover new therapeutic targets and reveal details about the underlying mechanisms of various diseases by integrating large genomic and protein datasets. More experimental research still needs to validate the anticipated hub genes and their functional roles in personalized medicine. We can improve our comprehension of and ability to treat periodontitis with systemic diseases by using machine learning to predict interactomic hub genes in peripheral blood mononuclear cells.

## Data Availability

https://www.ncbi.nlm.nih.gov/geo/query/acc.cgi?acc=GSE156993.
